# Assessment of parents´ knowledge regarding phenylketonuria and its affecting factors: a cross-sectional study

**DOI:** 10.11604/pamj.2022.41.308.25936

**Published:** 2022-04-14

**Authors:** Fatma Üneşi Öztürk, Selda Fatma Bülbül, Ayşegül Alpcan

**Affiliations:** 1 Kirikkale University School of Medicine, Department of Pediatric Metabolic Diseases and Nutrition, Kirikkale, Turkey

**Keywords:** Phenylketonuria, parents, information level, disease outcome, diet

## Abstract

**Introduction:**

the management of phenylketonuria (PKU) is complex. Practical skills and knowledge of individuals taking care of PKU patients are important for treatment compliance. This study investigated parents´ knowledge about PKU and its affecting factors. The study group consisted of 62 parents of PKU patients.

**Methods:**

data were collected using a ready-made questionnaire on sociodemographic characteristics, PKU and dietary treatment. Total knowledge score (KS) was calculated by giving 5 points for each correct answer.

**Results:**

the study included 34 mothers and 28 fathers; 90.3% of patients were diagnosed during the newborn screening program, 6.5% between ages 1 and 2, and 3.2% at age 6 or after. Among all, 38.7% presented to their follow-up appointments with their mothers, 61.3% with both their parents and none with their father alone. Mothers answered all questions more accurately than fathers. Mothers´ and fathers´ mean KSs were 73.97 ± 12.72 and 53.04 ± 22.25, respectively. The highest KS was obtained among parents whose children were 13 years of age or older.

**Conclusion:**

the presence of another family member, parents´ education level, working status and professional qualifications, previous training on PKU and family´s economic status were the affecting factors. Creating a PKU dietary plan requires knowledge and diligence. Patients and their parents should be periodically informed about the disease and dietary treatment in order to increase their level of knowledge.

## Introduction

Phenylketonuria (PKU) is an inherited metabolic disorder caused by a deficiency of phenylalanine (Phe) hydroxylase activity. This disease shows an autosomal recessive transition and constitutes the most common inherited disorder of amino acid metabolism [1, 2]. A high incidence is reported in Turkey due to high consanguinity within the population. While the incidence is generally 1/10000-15000 live births, it is even higher in Turkey (1/4000-5000 live births) [3, 4]. Neurological problems such as mental impairment, behavioral problems, epilepsy, and mental retardation may occur due to increased Phe levels in patients with late diagnoses [5]. Newborn screening programs could identify the children with PKU as early as possible, and the most crucial consequence of the disease, mental motor retardation, could be prevented with appropriate treatment. In the 1950s, Bickel and colleagues [6] reported that a Phe-restricted diet gives patients a chance of normal life and allows normal brain development. When a specific Phe-restricted diet and other necessary treatments, such as micronutrient supplementation, are started, symptoms begin to diminish [7]. Children with PKU who properly manage their diet usually do not show any complaints and grow normally. However, the desirable outcome would not always be achieved. Therefore, factors affecting development and diet compliance have been sought since the 1950s [8, 9].

Applying a limited diet is quite difficult due to intensive diet training, self-confidence, and self-sufficiency requirements. Dietary compliance of patients with PKU is completely under parental supervision throughout infancy. In childhood, parents play a significant role in diet adaptation and nutrient selection [10]. Caring for these children requires great attention and effort. Therefore, parents´ motivations, good understanding, and organizing and accepting the diet are vital factors for the surveillance of the diet [11]. Therefore, the management of children with PKU should be ensured by their families, nutritionists, and metabolic specialists [12]. The effect of knowledge and skills of PKU patients and their parents on diet perception cannot be underestimated. It is necessary to give regular training to patients and their parents about disease and diet to control the dietary intake and to ensure their adaptation to dietary treatments [13]. There are few studies on the effect of parent and patient knowledge on nutritional compliance and the treatment of PKU. Therefore, this study aimed to evaluate the knowledge levels of the parents of children with PKU residing in Anatolia, Turkey.

## Methods

**Setting and study design:** this cross-sectional study was conducted between May 2016 and March 2018 with 62 parents of PKU patients living in Turkey after the approval of the local ethical committee (Kirikkale University School of Medicine Ethical Committee) with a decision number of 10/10, April 27, 2015 (ethical consideration).

### Sampling strategy

According to the information obtained from the PKU Family Association, there are a total of 315 patients with PKU, 300 in Ankara and 15 in Kirikkale. A special sampling method was not used to form the research group. The study participants were inhabitants of different parts of Kirikkale and Ankara, managed in different specialist metabolic clinics in both cities. The study group consisted of the patients and their parents who applied to the Kirikkale University School of Medicine Hospital, Ankara Children´s Health and Diseases Hematology Oncology Training and Research Hospital, Child Nutrition and Metabolism Outpatient Clinic, and PKU Family Association and agreed to participate in the study. We could not reach most of the families due to the lack of contact information, and some parents (42/315) did not agree to participate. Finally, we performed this study with 62 patients (62/315), 19% of the whole population.

### Data collection

After providing detailed information about the study, signed informed consent was obtained from parents. The data were obtained by filling out a questionnaire with a face-to-face interview technique. In the second stage, the parents were informed about completing the research scale to be used in the study, and they did under the supervision of the researcher. The questionnaire was prepared by the researcher after a comprehensive literature review, having 10 questions on sociodemographic characteristics and 20 questions on PKU disease and diet. The questions on knowledge were based on the textbook Classical Pediatrics by Nelson [14], and the correct and incorrect answers were evaluated according to the information given in this textbook. The total knowledge level score (KLS) was calculated by giving 5 points for each correct answer and was given between 0 and 100 points (quantitative variables).

### Statistical analysis

The data were transferred to a computer with the SPSS 16.0 program (USA). Descriptive statistical analyses were performed. The values are presented as mean ± SD or median (min-max) and number (percentage). The distribution normality was tested using the Shapiro-Wilk test. The comparisons between continuous data were made with the Mann-Whitney U test, and the comparisons between categorical data were performed using the Chi-square test and Fisher exact test where applicable. A p-value of < 0.05 was considered statistically significant.

## Results

A total of 34 mothers (27.4%) and 28 fathers (22.6%) were included in the study. The mean ages of mothers, fathers, and children were 34.53 ± 7.22, 37.21 ± 6.27, and 9.65 ± 4.92 years, respectively (Table 1). More than 45% of the parents had less than 8 years of schooling. As the family´s socioeconomic status was concerned, more than 75% of the 62 parents described their family´s economic conditions as poor and/or average (Table 1). Among all, 46.8% of the parents were found to be married to relatives. Also, 56 (90.3%) children were diagnosed during the newborn screening program, 4 (6.5%) children were diagnosed between 1-2 years of age, and 2 children (3.2%) had the diagnosis at ages 6 years or more, and 87.1% of the families did not have any other children with PKU. To the question “Does your child go to controls regularly?” 17.7% (11/62) of the parents answered *“No and/or from time to time*.” It was learned that 61.3% (n = 38) of the children went to controls both with their mothers and fathers, 38.7% (n = 24) only with their mothers, and none only with their fathers.

**Table 1:** characteristics of PKU patients and the attitutes of the parents

		N	%
The age of child's diagnosis	In the scanning program	56	90.3
	1-2 age	4	6.5
	6 age and over	2	3.2
Kinship status	No	33	53.2
	2 degrees	23	37.1
	1st degree	6	9.7
Are there other childeren with PKU in the family?	No	54	87.1
	1 child else	8	12.9
Do you take your child regularly to check?	No/ Sometimes	11	17.7
	quarterly	33	53.2
	once in a month	13	21
	once a week/ biweekly	5	8.1
In general, who is going to control with child?	Mother	24	38.7
	Father	0	0
	Mother and father	38	61.3
Does the child take medication?	No	31	50
	Kuvan	7	11.3
	Neophe/protein powder	4	6.5
	Vitamin/fish oil	11	17.7
	I don´t know	9	14.5
Have you ever received a training on PKU or about the PKU diet?	Yes	48	77.4
	No	14	22.6

Note: PKU=Phenylketonuria

Among all, 48 parents (77.4%) stated that they had received some training on PKU and/or PKU diets (Table 2). Surprisingly, 14.5% (n = 9) of the parents did not know what medication their children were taking. Overall, maternal knowledge on most aspects of PKU and its diet was better than the fathers´. Mothers answered all questions more accurately than fathers, but the statistical difference was not significant (p > 0.05) (Table 3). On the other hand, the answer difference between the mothers and the fathers for the questions on PKU diet was more evident. The mothers´ correct answer rates were higher than fathers´ in all questions (Table 3). When we compared the answers of parents, the biggest difference between the mothers and fathers was observed in questions related to PKU diet, such as “Can breast milk be given to the baby with PKU?” How many meals should a child with PKU be fed? and “Which of the following foods can be consumed instead of bread?”

**Table 2:** the correct answers of the study group to the questions about PKU disease and dietary treatment

Questions		Mother		Father		Total		P
		N	%	N	%	N	%	
1) What is the cause of PKU?	True	31	91.2	25	89.3	56	90.3	1
2) Which enzyme is missing in PKU?	True	26	76.5	16	57,1	42	67.7	0.178
3) Why is the treatment of PKU disease important?	True	32	94.1	23	82.1	55	88.7	0.228
4) What is the most important treatment method in PKU?	True	34	100	26	92,9	60	96.8	0.201
5) When should the diet be started in PKU?	True	34	100	22	78,6	56	90.3	**0.006**
6)Can the normal level of intelligence be achieved when the right diet therapy is applied from birth?	True	33	97.1	22	78.6	55	88.7	0.039
7) What is the likelihood that a PKU carrier mother and father will be a PKU child again?	True	24	70.6	16	57.1	40	64.5	0.404
8)How many mg / dL of phenylalanine should normally be in the blood?	True	17	50.0	15	53.6	32	51.6	0.981
9) How long should the diet be used in PKU?	True	33	97.1	26	92.9	59	95.2	0.565
10) Can breast milk be given to a PKU baby?	True	29	85.3	13	46.4	42	67,7	**0.003**
11) How many meals a PKU child should eat per day?	True	24	70.6	11	39.3	35	56.5	0,027
12) What is the concept of change in diets?	True	26	76.5	10	35.7	36	58.1	**0.003**
13) Which of the following can be consumed instead of bread exchange?	True	32	94.1	12	42.9	44	71	**0.0001**
14) Which of the following is restricted to diet?	True	25	73.5	11	39.3	36	58.1	**0.014**
15) How many grams of phenylalanine (12.5 g) is there in a change bread?	True	17	50	8	28.6	25	40.3	0.147
16) How many mg of vegetables contain phenylalanine?	True	15	44.1	6	21.4	21	33.9	0.108
17) How many grams of margarine / butter (5 kg) contains phenylalanine ?	True	9	26.5	5	17.9	14	22.6	0.616
18) How many grams of an exchange fruit contains **phenylalanine?**	True	12	35.3	7	25	19	30.6	0.551
19) Which of the following is not limited to diet?	True	25	73.5	9	32.1	34	54.8	**0.003**
20) How many gr / pieces of olives can be consumed instead of an exchange of butter (5 g)?	True	30	58.8	9	32.1	29	46.8	0.066

**Table 3:** knowledge level scores (KLS) distributions according to the socio-demographic characteristics of parents

		Knowledge Level Scores						
Age		**n**	**X**	**Median**	**Min**	**Max**	**SD**	**P**
	1-3 age	5	63.00	75.00	35.00	80.00	21.39	0.816
	4-6 age	12	60.42	70.00	15.00	85.00	24.44	
	7-9 age	14	66.43	75,00	20.00	90.00	20.23	
	10-12 age	115	61.33	60.00	30.00	90.00	20.04	
	13-15 age	11	70.00	80.00	35.00	95.00	21.79	
	15 age and over	5	68.00	70.00	50.00	80.00	11.51	
Educational level	Low	28	55	55,00	15.00	80.00	19,10	**0.001**
	Middle	24	70.21	75.00	25.00	95.00	20.56	
	High	10	77.50	80.00	55.00	85.00	9.79	
Who answered the survey	Mother	34	73.97	75.00	40.00	95.00	12.72	**0.0001**
	Father	28	53.04	52.50	15.00	85.00	22.25	
Training on diet and disease	Yes	48	73.13	75.00	30.00	95.00	13.19	**0.0001**
	No	14	35.00	35.00	15,00	55.00	10.74	
Who is going to control with the child?	Only mother	24	56.67	67.50	15,00	90.00	22.44	0.016
	Mother with father	38	69.47	75.00	30.00	95.00	17.58	
Economic status	Good	15	3.33	80.00	35.00	95.00	17.49	**0.004**
	Average	27	68.15	75.00	15.00	90.00	19.22	
	Poor	20	53.00	55.00	20.00	85.00	19.70	

### Knowledge level score

KLSs according to the sociodemographic characteristics of the parents are shown in Table 3. Mothers´ and fathers´ mean KLSs were 73.97 ± 12.72 and 53.04 ± 22.25, respectively, and the difference was statistically significant (p < 0.05). When compared with children´s age, the highest KLS was obtained among the parents whose children were 13 years or more. KLSs showed a significant difference among the education levels of parents, so the KLSs of the primary school group were significantly lower than those of the other groups (p < 0.05). The KLSs of those who had previously received training on PKU and its diet were significantly higher than those of the parents who had not received any (75 ± 13.19 vs. 35 ± 10.74, respectively, p < 0.05). Mothers and fathers who were going to controls together showed higher KLSs than only mothers who went to controls (p < 0.05). Parents having more than one PKU patient at home showed higher KLSs than those who did not (75.63 ± 17.61 and 62.87 ± 20.43, respectively, p > 0.05).

## Discussion

Results of this study revealed that parents´ knowledge of the general issues of PKU was nearly perfect. However, when it accounted for the diet-related questions, the knowledge fell sharply (Table 3). For example, the questions that had 100% correct responses were “What is the most important treatment method in PKU?” and “When should the diet be started in PKU?” However, only 22.6% of the parents responded correctly to the questions on quantities of Phe in foods such as butter/margarine. The obtained results are in agreement with the data acquired in studies on mothers of children with PKU, which demonstrated poor parental dietary knowledge [12-15]. These results suggest that families may be more ignorant, especially on diet-related issues. This may be due to a low perception of risk about the disease. In addition, our results also point to the gap in the training programs on PKU and its treatment. There is a need to develop interactive educational programs regarding the Phe content in foods as less than 50% of the parents knew the Phe content in the most commonly used foods, such as butter, vegetables, or fruit (Table 3). It is obvious that early diagnosis of PKU and adaptation to its diet as early as possible could prevent permanent brain damage, where patients could maintain a normal life and would have the chance to complete their school career [16]. This depends mostly on the quality of blood Phe control, which is influenced highly by the quantity and frequency of protein substitute administered, the amount of natural protein eaten, and the adequacy of energy intake from low Phe nutrients. However, in small children, all these factors are dependent on parental ability and discipline to continually apply this special dietary regimen. Naturally, the ability of parents to follow such a rigorous regime and treatment plan would be affected by various factors. Among all other factors, parents´ insufficient knowledge of the disease and lack of parental educational achievement appear to be important in overall blood Phe control [17, 18].

Diets applied to patients with all metabolic diseases are difficult and require special attention. Parents who have children with PKU experience difficulties in learning and applying the required diet. All the parents do not have the same ability to learn the management of the diet; therefore, the nutritional treatment of the family should be arranged according to the socioeconomic status, educational learning desire, and diet therapy. Family support and the practical applications of PKU diet in training programs increase the success rate [10, 12]. Therefore, the parents need not only training at the time of diagnosis but also support by continuous lifelong visual and practical training. Besides, the child´s growth rate and blood Phe level should also be monitored periodically [12]. Mostly, parents are trained during their routine visits to the hospital. As shown in our study, usually mothers are the ones who go to controls with the children, and naturally, it has been expected that mothers would have significantly higher knowledge of the disease and the diet than fathers. However, there was no statistically significant difference for KLS between the mothers and fathers who went to controls with their children. KLSs were higher among mothers in our study group.

In this study, 74% of the families stated that they had received some training on PKU and the restricted diet. This result shows the inadequacy of training because every mother and father must have a diet education, and this rate should be 100%. To comply with the treatment in PKU, cooperation among doctors, dieticians, nurses, patients´ family and relatives, and patients is required. Training should be done to evaluate the level of knowledge of patients and their relatives at different times [19, 20]. Moreover, there is a need to form support groups and family therapy programs to overcome negative attitudes toward the PKU diet. This concerns the feeling of shame evoked by the necessity to follow a different diet. As mentioned by Rahgoi *et al*. [19] in a recent article, family-centered empowerment programs emphasizing an effective role of the family in the motivation, psychological growth, knowledge, attitudes, and perceived threats of the members can lead to health promotion.

In families with a child having a chronic illness, the care of the child and the workload of the hospital are mostly on the mother´s shoulders. This is not a cultural issue as Varni´s study [20] has shown that 80% of the parent forms were filled out by their mothers. As Raina *et al*. [21] reported that children with cerebral palsy are mostly nursed by their mothers, we understand that not only at the hospital but also at home, mothers undertake the major part of the sick child´s responsibility in all aspects of life. We have also seen that most of the children in our study were going to controls with their mothers (61.3%), and mothers gave more accurate answers to the questions on the specific diet regimen than fathers. Similarly, mothers´ KLSs were significantly higher than those of fathers. Therefore, the observed results are not surprising as mothers are more interested in their children´s diet and care. It should be emphasized that not only the mothers but also the fathers and/or other family members who care for the sick children should be taken into consideration when training programs are designed.

As mentioned before, the ability of parents to follow such a rigorous way would be affected by various factors. Our results revealed that the knowledge levels of the parents on PKU and its diet treatment decreased as the education level of the parents decreased. Similar to our results, Küçük Kasap [22] also observed a decrease in the dietary knowledge scores as the education level of the family decreased. Therefore, it would be appropriate to prepare the material to be used in the training programs according to the educational and cultural level of each family, and training should be continued until the trainers are sure that the family members fully understand the diet application. To control their diets in the adolescent period and to ensure their adaptation to dietary treatment, regular education programs on diseases and diets should be developed for parents and adolescents [14, 15]. Durham-Shearer *et al*. reported that adolescent and adult patients with PKU are aware of their dietary recommendations and are compatible with the diet [23]. In our study, the highest KLSs were obtained among the parents whose children were 13 years of age or older. Naturally, the information gained about the disease increases over the years.

**Limitations:** our study has the following limitations. First, we analyzed the questionnaire results, and this does not reflect the main status of the patients. Second, these results belong to the limited geographic area in Turkey and could not be generalized for the whole country.

## Conclusion

In conclusion, we observed that the level of knowledge of the parents about the disease and diet therapy is insufficient. To effectively care for PKU patients, special dietary attention and knowledge of the disease are required; otherwise, there will be a lot of negligence and error in treatment. For this reason, the patients and their family (both parents together) should be informed about the disease and diet therapy at specific intervals. Nutrition education should be provided to both parents according to the educational status and living conditions of the family. These trainings should be given by nutritionists who are trained primarily in metabolic diseases. This study confirms the need for professional actions to impart the practical knowledge of PKU nutrition to the family. Moreover, there is a need to form support groups and family therapy programs to overcome negative attitudes toward the PKU diet. This concerns the feeling of shame evoked by the necessity to follow a different diet.

### What is known about this topic

- Newborn screening programs could identify the children with PKU as early as possible, and the most crucial consequence of the disease, mental motor retardation, could be prevented with appropriate treatment.

### What this study adds

- This study shows the need for professional actions to impart the practical knowledge of PKU nutrition to the family;
- Moreover, there is a need to form support groups and family therapy programs to overcome negative attitudes toward the PKU diet;
- This concerns the feeling of shame evoked by the necessity to follow a different diet, especially in adolescents.
